# The clinical significance of binge eating among older adult women: an investigation into health correlates, psychological wellbeing, and quality of life

**DOI:** 10.1186/s40337-022-00621-x

**Published:** 2022-07-07

**Authors:** Lisa Smith Kilpela, Victoria B. Marshall, Pamela K. Keel, Andrea Z. LaCroix, Sara E. Espinoza, Savannah C. Hooper, Nicolas Musi

**Affiliations:** 1 Barshop Institute, UT Health San Antonio, San Antonio, TX USA; 2 ReACH Center, UT Health San Antonio, San Antonio, TX USA; 3 South Texas VA Health System, Audie Murphy Veterans Hospital, San Antonio, TX USA; 4 Department of Psychology, Florida State University, Tallahassee, FL USA; 5 Herbert Wertheim School of Public Health and Longevity Science, University of California San Diego, San Diego, CA USA

**Keywords:** Older adults, Binge eating, Women’s health, Health-related quality of life

## Abstract

**Background:**

One type of overnutrition, binge eating (BE; eating an unusually large amount of food with loss of control), is prevalent among older adult women. Yet, little is known about the clinical significance of this eating disorder pathology in older adults, especially in relation to health outcomes used in geriatrics, while controlling for associations with body mass index (BMI).

**Method:**

Women (*N* = 227) aged 60–94 completed two measures of BE and health/wellness questionnaires online. We used multivariable analyses to compare women with Clinical-frequency BE (≥ weekly frequency), Subclinical-frequency BE (< weekly), and No BE on health/wellness outcomes controlling for BMI. We conducted partial correlations controlling for BMI to examine associations between BE severity and health indices.

**Results:**

Controlling for BMI, the Clinical-frequency BE group reported poorer health-related quality of life (physical function, role limitations due to both emotional and physical problems, vitality, emotional wellbeing, social function, and pain) and poorer psychological health (depression, body image) compared to both Subclinical-frequency BE and No BE. The Clinical-frequency BE group also reported poorer sleep, nutritious food consumption, general health, and positive affect compared to No BE. Associations between a separate measure of BE severity and health indices confirmed findings from group comparisons.

**Conclusion:**

Weekly BE may offer a promising screening benchmark for identifying one type of overnutrition in older women that is associated with numerous indicators of poorer health, independent of the effects of BMI. More research is needed to understand risks for and consequences of BE unique to older adult women.

**Plain English Summary:**

Binge eating (BE; eating an unusually large amount of food with loss of control), is prevalent among older adult women and is associated with health problems in younger populations. Yet, little is known about how BE is related to other health problems in older adults. We compared health behaviors, physical health, health-related quality of life, and psychological health between older adult women who reported weekly or more frequent BE (i.e., Clinical BE), those with low frequency BE (i.e., Subclinical BE), and those with no BE, while accounting for BMI. Older women in the Clinical BE group reported poorer health-related quality of life, more depression symptoms, and worse body image compared to the Subclinical BE and No BE groups. Compared to the No BE group, the Clinical BE group also reported poorer sleep, less frequent consumption of nutritious foods, worse health, and less frequent positive emotions. Using a separate measure of BE severity, we found similar associations with these health outcomes. Engaging in weekly BE may represent one type of overnutrition behavior in older women that is associated with numerous indicators of poorer health. More research is needed to understand risks for and consequences of BE unique to older adult women.

## Background

Eating disorder pathology, including both under-and over-nutrition, can negatively affect healthy aging. One form of overnutrition is binge eating (BE), which refers to discrete episodes of consuming an abnormally large amount of food in one sitting while simultaneously feeling out of control [1]. Historically viewed as a problem of youth [2, 3], BE remains relatively understudied in older adult populations. Yet, recent survey research indicates that 3.5–12% of women in midlife (age 50+; [4, 5]) and older (age 60+; [6, 7]) engage in recurrent BE; while 19% of women aged 65–90 reported subjective BE in the past month [7]. Additionally, 5.6% of women aged 65–94 reported at least one objective or subjective BE episode in the past month, with a mean frequency of 8 episodes/month for objective binge episodes, when evaluated by structured clinical interview [8]. Thus, older adult women suffer from recurrent BE at a higher rate than previously thought [9, 10]. However, little is known about the experience of older women with BE, especially in the context of geriatrics-relevant health indices.

Of note, BE is associated with numerous mental and physical health problems in the general population. Binge Eating Disorder (BED; a disorder characterized by weekly BE) is closely linked with obesity [11] and is associated with poor macronutrient intake, as BE episodes typically involve consumption of foods high in sugar, fat, and salt [12]. Beyond obesity and poor nutrition, BED is associated with medical comorbidities including cardiovascular issues, metabolic syndrome, sleep disturbance, and pain [13, 14], and BED can affect blood glucose levels and impact diabetes management [15]. Furthermore, individuals suffering from BED experience higher rates of psychiatric disorders, insomnia, and lower life satisfaction [14, 16]. Recurrent BE (including both clinical and partial BE syndromes; [17]) is associated with psychosocial impairment and comorbid psychopathology in younger samples [17, 18]. Even when controlling for comorbid psychiatric disorders, both recurrent BE and BED are associated with higher rates of diabetes, gastrointestinal problems, menstrual complications, somatic symptoms, and disability [13, 14].

Among older populations, the consequences of BE may be severe, as obesity and depression often exacerbate existing medical morbidities common among older adults [19]. Nonetheless, older adults have been consistently excluded from BE research [20, 21], leaving the clinical significance of recurrent BE in this population poorly understood. In the limited research to date, findings suggest that BE is related to greater depressive symptoms, higher BMI, poorer body image, and additional ED symptoms among midlife women [5, 22]. A study of midlife and older adult women (aged 46–76) found that overall disordered eating was strongly associated with depression and with less physical activity [23]. Among older women (aged 60–75), BE frequency was positively correlated with higher BMI, age-appearance anxiety, and eating concerns [24]. In a small study of older women (aged 65–77) with BED, participants reported an average BE frequency of 4.5 (± 2.9) episodes per week; depression, anxiety, and elevated BMI were highly comorbid [21]. Notably, most research on BE among midlife and older samples has been limited to psychological comorbidities and BMI, while research investigating health correlates of recurrent BE in younger populations has been more extensive. Thus, both common and unique comorbidities among older and younger populations remain largely unknown.

Although the vast majority of research regarding BE includes younger women, experiences related to the aging process may constitute risk for this eating disordered behavior among older women. Specifically, the menopausal transition has been hypothesized as a time of increased risk for BE [9, 25]. Hormone fluctuations hallmark of the menopausal transition have been associated with BE frequency in perimenopausal women [26]. Sleep disturbances and negative affect common during the menopausal transition [27] are well-established risk factors for BE. Furthermore, psychosocial stressors that are more common as women age (e.g., divorce or marital conflicts, caregiving demands, empty nest syndrome, interpersonal loss), may constitute risk for dysregulated eating behaviors [28–30]. Indeed, research suggests that ED cases in older adulthood may be a combination of new onset, chronic/lifelong, or remit/relapse cases [31], with ranges of late-life ED onset ranging from 28 to 69% [31, 32]. Thus, the exclusion of older women from eating disorders research and exclusion of eating disorders from research in older women does not reflect risk for BE and associated health consequences in this group. Related to this, there are limited data on the relation of BE symptomatology and health outcomes particularly relevant in geriatrics, such as physical dysfunction, role limitations, sleep quality, and social functioning, in older populations.

### The current study

Efforts to understand the intersection of aging and BE are needed, as prevalence rates of BE among older women are high while health correlates of BE in this population remain largely unknown. Therefore, the present study sought to examine the frequency and health correlates of BE in women aged 60 and over. According to the current Diagnostic and Statistical Manual for Mental Disorders (DSM-5), BE once a week over the past three months meets the frequency criterion for a diagnosis of binge eating disorder [1]. This cutoff, however, was identified using clinical data from the general adult population, which predominantly includes young adults and did not include data from older adults [1].

Therefore, our first aim was to investigate if recurrent BE (defined per the DSM-5 frequency criterion) was associated with poorer health/wellness outcomes, indicating a clinically relevant threshold among older adult women (age 60+). To accomplish this aim, we compared health/wellness indices across three groups of older adult women based on symptom frequency: (1) No BE, (2) Subclinical BE (i.e., < weekly), and (3) Clinical BE (i.e., ≥ weekly). We predicted that women in the Clinical BE group would have poorer health indices than those in both the Subclinical BE and No BE groups. To ensure that associations reflected the presence of BE rather than elevated BMI, we controlled for BMI in all analyses. Our second aim was to further probe the relation of BE symptom severity and health/wellness indices using a separate measure of BE severity, again controlling for BMI. We hypothesized that greater BE severity would be correlated with poorer health/wellness indices in this sample of older adult women. Importantly, several of these health indices have not been examined in prior studies given their unique relevance to older participants.

## Method

### Participants

Participants included 227 women aged 60–94 years (*M* = 68.84, SD = 6.53). Regarding race/ethnicity, 91.6% endorsed white race and 14.1% endorsed Hispanic ethnicity (see Table 1). The sample was highly educated with 67.8% reporting a bachelor’s degree or higher. Of the participants who reported their relationship status, 54.6% reported being married or cohabitating, and 100% were community-dwelling.

**Table 1:** Participant demographics and baseline characteristics (N = 227)

Measures	No BE (*n* = 118) *M* (SD) or *N* (%)	Sub-Clinical (*n* = 68) *M* (SD) or *N* (%)	Clinical (*n* = 41) *M* (SD) or *N* (%)
Age	69.10 (6.83)	69.40 (6.09)	67.15 (6.19)
BMI	28.22 (6.89)	28.87 (6.82)	33.26 (10.49)
Race			
White	112 (94.9%)	59 (86.8%)	37 (90.2%)
Black or African American	1 (0.8%)	2 (2.9%)	2 (4.9%)
Asian	–	2 (2.9%)	–
Native American/Alaskan Native	–	1 (1.5%)	–
Other or mixed race	3 (2.5%)	3 (4.4%)	–
Ethnicity			
Hispanic/Latina	12 (10.2%)	9 (13.2%)	11 (26.8%)
Non-Hispanic/Latina	103 (87.3%)	57 (83.8%)	29 (70.7%)
Relationship Status			
Married/living with partner	63 (53.4%)	37 (54.4%)	24 (58.5%)
Education			
Bachelor’s degree or more	81 (68.7%)	43 (63.3%)	30 (73.2%)

### Procedure

The Institutional Review Board (IRB) deemed this study as IRB-exempt (i.e., no more than minimal risk to study participants). Older adult women aged 60 + years were recruited via internet advertising using various strategies. We recruited publicly online (e.g., social media sites, interest groups for older adult women, local senior centers, and institutional websites), snowball sampling (i.e., asking women to forward the survey to their networks), Amazon Mechanical Turk, and by word of mouth (e.g., Community Advisory Board meetings). Online surveys are commonly used in studies with older adults, including those within the ED field [22, 33], and current data confirm that 75% of adults age 65 + use the internet [34]. Recruitment materials advertised a study on “women’s health and aging” to reduce bias in prevalence estimates and correlations. After consent, participants completed self-report questionnaires online. All measures were either developed for, validated in, or commonly used in older adult samples. Upon completion, participants had the option to provide an email address to enter a raffle for a US $50 Amazon e-gift card.

We initially launched the survey offering participants a $10 Amazon e-gift card. Careful monitoring of data revealed an influx of artificial responses (i.e., non-human “bots”), which has become a prevalent problem online survey studies encounter [35]. We discarded over 400 artificial responses collected within less than 24 h, embedded validation questions into the survey, added “dummy” pathways for potential respondents who were either younger than 60 years of age or who identified as male gender, and changed to a raffle system to reduce the likelihood of “bot” responses. Only responses from eligible participants that passed validity checks (e.g., checks for unreasonably short completion time, invalid text, repeated entries, response biases indicating inattention or not reading) were included in analyses. Of note, all validity checks would have screened out any invalid responses due to severe cognitive impairment.

### Measures

*Demographics* Participants reported age, race/ethnicity, highest level of education, annual household income, living arrangement, relationship status, employment status, medical/psychiatric history, history of any kind of eating disorder, and frequency of compensatory behaviors in the past 28 days.

#### Binge eating (BE)

*BE frequency* To assess frequency of current BE, we used the VA-Binge Eating Screener (VA-BES; 36), which asks: “On average over the past few months, how often have you eaten extremely large amounts of food at one time and felt that your eating was out of control at that time?” Response options include: Never, < 1 time/week, 1 time/week, 2–4 times/week, and 5 + times/week. This measure demonstrated good psychometric properties in prior samples with older adults [36]. In the current study, it demonstrated concurrent validity with our second measure of BE, described below.

*BE severity* We used the 16-item Binge Eating Scale (BES; [37]) to assess the behavioral manifestations of and thoughts/feelings associated with BE. Each item on the scale consists of four statements that reflect severity (0 indicates no BE problem and 3 indicates a severe BE problem). Items are summed for a final score; higher scores indicate greater BE severity. The BES has demonstrated good psychometric properties in past research, and internal consistency in the current sample was high (Cronbach’s α = 0.90). In addition, BES scores were significantly different between the BE frequency groups, controlling for BMI (*F*(2, 213) = 44.49, *p* < 0.001). The Clinical BE group (*M* = 14.74, SD = 9.47) reported the significantly higher BES scores than the Subclinical BE group (*M* = 7.57, SD = 5.84, *p* < 0.001, 95% CI [0.24, 0.60]), which was significantly higher than the No BE group (*M* = 3.97, SD = 4.55, *p* < 0.001, 95% CI [0.10, 0.37]).

#### Health behaviors and physical health

*BMI and diabetes status* Participants self-reported current height/weight, and both current and past diagnoses of diabetes mellitus and pre-diabetes. Although self-report is not optimal for assessing height and weight, research indicates that self-report weights are reasonably accurate when direct measurement is not feasible [38].

*Nutritious foods* The two-item Eating Behaviors Questionnaire (EBQ; [39]) measured consumption of nutrient-dense foods (e.g., fresh fruits and vegetables). Items are rated on a 5-point Likert scale (1 = consume at every meal, 5 = never). The items are summed, and higher scores indicate less consumption of nutritionally dense foods (i.e., higher scores indicate less nutritious food intake). Internal consistency in the current sample was adequate (α = 0.65).

*Sleep quality* The Pittsburgh Sleep Quality Index (PSQI; [40]) assessed quality of sleep. The measure contains 19 self-rated questions that are combined to form seven component scores, each of which has a range of 0–3 points. These component scores are then summed to yield a global PSQI score, which has a range of 0–21 and a clinical cutoff of 5; higher scores signify worse sleep quality. Internal consistency in the current sample was good (α = 0.73).

*Alcohol use* We used the 3-item version of Alcohol Use Disorders Identification Test [41] to screen for possible alcohol misuse. Higher scores suggest greater use of alcohol, and a score of 3 or more is considered a positive screen for alcohol misuse among women. Internal consistency in this sample was low (α = 0.49). Thus, we did not proceed with inferential statistics using this measure.

*Physical activity* The Physical Activity Scale for the Elderly (PASE; [42]) measured level of physical activity. The scale consists of 21 items of self-reported occupational, household, and leisure activities over a one-week period. Total PASE scores were computed by multiplying the amount of time spent in each activity (hours per day over a 7-day period) by the respective weights and summing over all activities; higher scores indicate more physical activity. Internal consistency for this scale was adequate (α = 0.68).

#### Quality of Life (QOL)

*Health-related QOL* We used the RAND 36-Item Short Form Health Survey (SF36; [43]), which taps into eight health concepts: physical functioning, bodily pain, role limitations due to physical health problems, role limitations due to personal or emotional problems, emotional well-being, social functioning, energy/fatigue, and general health perceptions. The scores are weighted sums of the questions in each of the eight sections. Scores range from 0 to 100, with lower scores indicating more disability. Internal consistency statistics were good for all domains (α range = 0.79-0.91).

*Social isolation* We used the 8-item PROMIS Social Isolation scale [44] to assess perceptions of being avoided, excluded, detached, disconnected from, or unknown by, others. Items are rated on a 5-point scale (1 = never, 5 = always). Final scores are summed, and higher scores signify greater feelings of social isolation. This measure was added after data collection began; thus, the sample for this measure is smaller than for other measures (*n* = 97). Internal consistency for this sample was high (α = 0.96).

*Food insecurity* The 6 item USDA Short Form Food Insecurity Scale [45] assessed financially based food insecurity and hunger in households over the past 12 months. Final scores range from 0 to 6 with lower scores indicating a higher level of food insecurity (current sample α = 0.89).

#### Psychological health

*Depressive symptoms* We used the 10-item Center for Epidemiologic Studies—Depression Scale (CES-D; [46]) to measure depression. Items were rated on a 4-point scale (ranges from 0 = rarely or none of the time to 3 = all of the time). Scores are summed, and higher scores signify greater depressive symptoms (current sample α = 0.87).

*Anxiety* We used the Geriatric Anxiety Inventory Short Form (GAI; [47]) to detect anxiety symptoms. This scale contains 7 self-report items to measure anxiety symptoms over the past week. Items are rated “agree” or “disagree,” and scores are summed. Higher scores indicate greater anxiety symptoms. Internal consistency for this sample was high (α = 0.93).

*Positive emotions* The positive affect subscale of the Positive and Negative Affect Schedule (PANAS-X; [48]) assessed frequency of experiencing positive emotions over the past 3 weeks. Higher scores represent more frequency positive emotions, and internal consistency for this sample was high (α = 0.92).

*Body Image* To assess positive body image, we used the Body Appreciate Scale (BAS; [49]), which is a 10-item scale assessing experiences of respect towards or appreciation of their body (e.g., “I feel good about my body”); higher scores indicate more positive body image. Internal consistency for this sample was high (α = 0.95).

*Grief* We used a 6-item grief symptom composite scale short form (based on Boerner et al., 2005; [50]) to assess the experience of grief in the event of losing a loved recently. Participants who selected “yes” to having lost a loved one in the past 6 months answered questions about grief experiences. Internal consistency in this sample was good (α = 0.81).

#### Data analyses

Nearly all participants (89%) completed all survey measures, and all available data were used in analyses. No significant differences were observed between those who did and did not complete all survey measures on demographic or clinical variables (all *p* values > 0.10). For multi-item measures with at least 10 items that do not utilize transformations in the scoring process (e.g., SF-36, PSQI, PASE), we imputed missing values by using mean item substitution with a criterion of 75% of items completed. Measures that met these criteria were: BES, CES-D, PROMIS Social Isolation, PANAS, and BAS.

For our first aim, we conducted planned contrasts of the Clinical BE group versus the Subclinical BE and No BE groups using a MANCOVA, controlling for BMI, to control for family-wise error rate. Although there has been some debate regarding the interpretation of planned comparisons based on the significance of the omnibus test, we followed guidelines detailed by Furr and Rosenthal [51]. These guidelines explain the relevance of using a priori planned contrasts regardless of (and even instead of) omnibus *F* tests [51–53]. As a sub-aim of Aim 1, we explored possible differences between the Subclinical and No BE groups on key outcomes. Because these analyses were post hoc in nature, we used a Bonferroni correction to account for multiple tests and had no specific hypotheses. For our second aim, we conducted partial correlations between BES scores (i.e., BE severity) and health outcomes, while controlling for BMI.

## Results

Based on the VA-BES, *n* = 118 (51.98%) participants were in the No BE group, *n* = 68 (29.96%) were in the Subclinical BE group, and *n* = 41 (18.06%) were in the Clinical BE group. Our three-group analyses of variance indicated a between-groups difference for BMI (*F*(2, 219) = 6.49, *p* = 0.002). A bivariate Pearson correlation also confirmed that BE severity was positively correlated with higher BMI (*r* = 0.31; *p* < 0.001). Regarding purging behaviors, 1.7% (*n* = 4) reported any self-induced vomiting and use of laxatives/diuretics to control weight/shape in the past 28 days, while an additional 0.9% (*n* = 2) reported any laxative/diuretic use without vomiting to control weight/shape in the past 28 days.

### Aim 1 results

The overall MANCOVA model was significant (*F*(30,176) = 1.69, *p* = 0.02), therefore we examined differences between the three groups at each dependent variable (Table 2). For the purpose of reporting results, we grouped the dependent variables into three domains: health behaviors and physical health, health-related QOL, and psychological health. Omnibus results for each dependent variable, descriptive statistics, and effect sizes for between group comparisons are presented in Table 2.

**Table 2:** Outcomes as a function of binge eating frequency group and between-groups effect sizes

	No BE *n* = 118 *M* (SD)	Subclinical BE *n* = 68 *M* (SD)	Clinical BE *n* = 41 *M* (SD)	Omnibus *F*(df), *p*	Clin-sub *d*	Clin-no *d*	Sub-no *d*
*Health behaviors*							
EBQ	3.54 (1.37)^b^	3.85 (1.47)^ab^	4.24 (1.58)^a^	*F*(2,212) = 2.29, *p* = .103	.26	.47	.22
PSQI	6.39 (3.98)^b^	6.57 (3.42)^ab^	8.29 (3.66)^a^	*F*(2,184) = 2.27, *p* = .106	.49	.50	.05
PASE^†^	133.27 (85.07)	155.48 (114.14)	120.64 (91.70)	*F*(2,191) = 1.68, *p* = .189	.34	.14	.22
Current DM^‡^	6 (5.1%)	7 (10.3%)	5 (12.2%)	–	–	–	–
History DM^‡^	3 (2.5%)	1 (1.5%)	2 (4.9%)	–	–	–	–
Current Pre-DM^‡^	6 (5.1%)	4 (5.9%)	5 (12.2%)	–	–	–	–
History Pre-DM^‡^	13 (11.0%)	10 (14.7%)	10 (24.4%)	–	–	–	–
*Quality of life*							
Phys Function^†^	81.22 (21.70)^b^	79.52 (21.79)^b^	65.29 (26.04)^a^	*F*(2,201) = 4.06, *p* = .019*	.59	.66	.08
Limits Phys^†^	81.25 (33.16)^b^	78.63 (32.28)^b^	61.11 (40.29)^a^	*F*(2,202) = 3.75, *p* = .025*	.48	.55	.08
Limits Emot^†^	86.42 (26.59)^b^	86.02 (30.51)^b^	66.67 (38.21)^a^	*F*(2,202) = 5.56, *p* = .004*	.56	.60	.01
Wellbeing^†^	78.90 (16.52)^b^	79.68 (14.50)^b^	72.11 (17.40)^a^	*F*(2,205) = 3.19, *p* = .043*	.47	.40	.05
Social Function^†^	89.25 (18.21)^b^	89.48 (18.68)^b^	73.96 (25.07)^a^	*F*(2,202) = 8.38, *p* < .001*	.70	.70	.01
Vitality^†^	65.78 (20.47)^b^	64.84 (16.51)^b^	51.11 (21.42)^a^	*F*(2,204) = 6.24, *p* = .002*	.72	.70	.05
Bodily pain^†^	76.22 (21.31)^b^	76.98 (19.11)^b^	66.42 (22.92)^a^	*F*(2,205) = 2.81, *p* = .063	.50	.44	.04
General Health^†^	81.72 (16.32)^b^	78.89 (15.17)^ab^	72.00 (21.18)^a^	*F*(2,119) = 2.12, *p* = .125	.37	.51	.18
Social Isolation	14.04 (7.06)	16.00 (7.78)	17.37 (7.56)	*F*(2,87) = 1.36, *p* = .262	.18	.46	.26
*Psychological*							
CES-D	5.36 (5.48)^b^	5.73 (4.68)^b^	8.64 (5.54)^a^	*F*(2,203) = 5.20, *p* = .006*	.57	.60	.07
GAI	1.63 (2.79)	1.53 (2.21)	2.20 (2.44)	*F*(2,187) = .41, *p* = .662	.29	.22	.04
PANAS^†^	3.55 (0.77)^b^	3.49 (0.68)^ab^	3.21 (0.68)^a^	*F*(2,191) = 2.15, *p* = .120	.41	.47	.08
BAS^†^	4.02 (0.82)^b^	3.98 (0.76)^b^	3.25 (0.73)^a^	*F*(2,196) = 9.51, *p* < .001*	.98	.99	.05

*Clin-Sub* clinical compared to subclinical, *Clin-No* clinical compared to no BE eating, *Sub-No* subclinical compared to no binge eating, *EBQ* Eating Behaviors Questionnaire, *PSQI* Pittsburg Sleep Quality Index, *PASE* Physical Activity Scale for the Elderly, *Phys function* physical function SF-36 domain, *Limits Phys* role limitations due to physical problems SF-36 domain, *Limits Emot* role limitations due to emotional problems SF-36 domain, *Social Isolation* PROMIS Social Isolation scale, *CES-D* Center for Epidemiologic Studies – Depression Scale, *GAI* Generalized Anxiety Inventory Short Form, *PANAS* positive affect subscale of the Positive and Negative Affect Schedule, *BAS* Body Appreciation Scale

^†^Higher scores indicate better health, higher scores on all other scales indicate poorer health/more pathology

^‡^Fisher’s Exact test used to investigate differences, data presented are n (%)

*N* = 227

Superscripts that differ reflect significant differences (*p* < .05) between groups

*p < .05

#### Health behaviors and physical health

Results indicated that women in the Clinical BE group reported less frequent consumption of nutritious foods compared to the No BE group (*t*(212) = 2.02, *p* = 0.045), but not compared to the Subclinical BE group (*t*(212) = 0.99, *p* = 0.360). Women in the Clinical BE group also reported poorer sleep than the No BE group (*t*(184) = 2.07, *p* = 0.040), but not the Subclinical BE group (*t*(184) = 1.83, *p* = 0.069). There were no group differences in physical activity levels.

As another indicator of physical health, we inquired about current or past diagnosis of Diabetes Mellitus (DM) or pre-DM. A 3 × 3 Fisher’s Exact test (DM levels: never DM, history of DM, or current DM) indicated no significant differences across groups for self-reported DM diagnosis (*p* = 0.31). Similarly, there were no differences across groups for self-reported pre-DM (*p* = 0.12), per 3 × 3 Fisher’s Exact test. Frequencies are reported in Table 2.

#### Quality of life

Women in the Clinical BE group reported poorer physical function than those in both the Subclinical BE [*t*(201) = 2.41, *p* = 0.017] and No BE groups [*t*(201) = 2.78, *p* = 0.006] and greater role limitations due to physical problems than both the Subclinical BE group [*t*(202) = 2.20, *p* = 0.029] and the No BE group [*t*(202) = 2.70, *p* = 0.007]. The Clinical BE group also reported more role limitations due to emotional problems than both the Subclinical BE group [*t*(202) = 2.92, *p* = 0.004] and the No BE group [*t*(202) = 3.19, *p* = 0.002], as well as worse emotional wellbeing compared to both the Subclinical BE (*t*(205) = 2.34, *p* = 0.02) and No BE groups [*t*(205) = 2.31, *p* = 0.022]. Additionally, the Clinical BE group reported lower vitality than both the Subclinical BE [*t*(204) = 3.03, *p* = 0.003] and No BE groups [*t*(204) = 3.42, *p* < 0.001], more pain than both the Subclinical BE [*t*(205) = 2.22, *p* = 0.028] and No BE groups [*t*(205) = 2.14, *p* = 0.033], and poorer social functioning than both the Subclinical BE [*t*(202) = 3.66, *p* < 0.001] and No BE groups [*t*(202) = 3.86, *p* < 0.001]. Finally, the Clinical BE group reported poorer general health than the No BE group [*t*(119) = 2.05, *p* = 0.042], but not compared to the Subclinical BE group [*t*(119) = 1.37, *p* = 0.172]. Contrary to hypotheses, we found no group differences in social isolation. Regarding food insecurity, only 4.4% of our sample (*n* = 10) reported food insecurity; therefore, we did not continue with inferential statistics.

#### Psychological health

Women in the Clinical BE group reported significantly greater depression than both the Subclinical BE [*t*(203) = 2.62, *p* = 0.010] and No BE groups [*t*(203) = 3.18, *p* = 0.002], as well as lower positive affect than the No BE group [*t*(191) = 2.06, *p* = 0.040] but not the Subclinical BE group [*t*(191) = 1.59, *p* = 0.113]. Women in the Clinical BE group reported poorer body image than those in both the Subclinical [*t*(196) = 3.88, *p* < 0.001] and the No BE groups [*t*(196) = 4.17, *p* < 0.001]. Contrary to hypotheses, there were no differences in anxiety across groups. Only 12.3% (*n* = 28) reported having lost a loved one in the past 6 months; therefore, we did not proceed with inferential statistics examining grief.

*Exploratory (Sub-aim 1) results* As a post hoc investigation into possible group differences between the Subclinical BE and No BE groups, Bonferroni corrected pairwise analyses indicated there were no significant differences between these two groups on any outcome. Effect sizes are presented in Table 2.

### Aim 2 results

Table 3 presents partial correlations of associations between BE severity (BES scores) and health indicators, controlling for BMI. BE severity was significantly correlated with worse health behaviors (less consumption of nutritious foods and sleep), lower health-related QOL (greater role limitations due to physical and emotional problems, lower vitality, poorer emotional wellbeing, worse social functioning, and higher feelings of social isolation), and poorer psychological health (greater depression, less positive affect, poorer body image) while controlling for BMI. BES scores were not correlated with anxiety, physical function, pain, general health, or physical activity while controlling for BMI.

**Table 3:** Partial correlations between BE severity and health outcomes controlling for BMI

Variables	*r*	*p*
Health behaviors		
EBQ	.20	.002*
PSQI	.29	< .001*
PASE^†^	− .03	.326
Quality of Life		
Phys Function^†^	− .09	.106
Limits Phys^†^	− .12	.047*
Limits Emot^†^	− .12	.041*
Wellbeing^†^	− .31	< .001*
Social Function^†^	− .17	.009*
Vitality^†^	− .29	< .001*
Bodily pain^†^	− .09	.106
General Health^†^	− .10	.128
Social Isolation	.25	.010*
Psychological		
CES-D	.33	< .001*
GAI	.10	.093
PANAS^†^	− .28	< .001*
BAS^†^	− .54	< .001*

*EBQ* Eating Behaviors Questionnaire, *PSQI* Pittsburg Sleep Quality Index, *PASE* Physical Activity Scale for the Elderly, *Phys function* physical function SF-36 domain, *Limits Phys* role limitations due to physical health problems SF-36 domain, *Limits Emot* role limitations due to personal or emotional problems SF-36 domain, *Social Isolation* PROMIS Social Isolation scale, *CES-D* Center for Epidemiologic Studies – Depression Scale, *GAI* Generalized Anxiety Inventory Short Form, *PANAS* positive affect subscale of the Positive and Negative Affect Schedule, *BAS* Body Appreciation Scale

*N* = 225

^†^Higher scores indicate better health, higher scores on all other scales indicate poorer health/more pathology

*p < .05

## Discussion

The present study sought to examine the frequency and health correlates of recurrent binge eating (BE) in older adult women aged 60 and over. Specifically, we investigated the clinical relevance of weekly BE, which is the clinical level frequency criterion for binge eating disorder as defined by the DSM-5 [1]. Importantly, the data used to establish this clinical frequency criterion originated from the general adult population of which most studies use an upper bound age limit of 60 or 65 [54]. In the current sample with a mean age of almost 69 years, 18% reported engaging in BE weekly or more frequently over the past few months. This is consistent with recent research, which found rates of weekly BE ranging from 19 to 26% across three independent samples of older adult women [55]. Another study found that 12% of older women reported BE in the past month, while 19% reported episodes of loss-of-control eating [7]. Thus, the current prevalence rate is both in line with past survey research in this population and further supports the contention that BE is common among older women.

Our findings indicated that BE is not only prevalent (18%), but this disordered eating behavior is linked with indices of poorer healthspan in our sample. Indeed, women who reported weekly or more frequent BE (i.e., Clinical BE) reported worse health/wellness indices than those with low frequency (i.e., Subclinical BE) and No BE. Additionally, greater BE severity on a separate measure was associated with indices of poorer health/wellness. Associations cannot be attributed to elevated BMI as all analyses controlled for BMI. Thus, in addition to potentially contributing to worse health outcomes via contributing to excess weight over time, Clinical BE may directly contribute to worse health outcomes in this population. Exploratory analyses supported no significant differences between the Subclinical and No BE groups, suggesting that occasional BE may not require clinical attention. However, longitudinal studies should examine whether Subclinical BE transitions to Clinical BE over time.

In our study, only 4.4% (*n* = 10) of women reported a history of an eating disorder. This could mean that older women are mostly struggling with newer onset BE related to risk factors specific to their developmental stage (e.g., menopausal transition or role transitions). That said, past literature suggests that individuals are often inaccurate when it comes to recognizing their own eating disorder [56]. This lack of recognition contributes to not seeking treatment. Therefore, older adult women may have fallen through the cracks in receiving a diagnosis of BE earlier in life and BE behaviors have persisted over the life course. In this context, the stereotype of eating disorders as problems of youth may further interfere with self-recognition and treatment of clinical BE among the older adult population. Recent calls have been made to include universal screening for eating disorders [57]; however, efforts have focused almost exclusively on younger populations and educational settings (e.g., college students; [58]). Our data indicate the importance and utility of truly universal screening that would detect weekly BE in older women. Given that most of these women would be encountered in primary care or geriatric health care settings, adding questions used in the current study to routine intake medical screens could identify women who require intervention.

Overall, our findings are consistent with prior research in the general population suggesting that clinical levels of BE are associated with distress and impairment, including poorer health-related QOL [18, 54, 59–61]. Of note, constructs within health-related QOL (e.g., physical function, social engagement, vitality) are particularly important to evaluate as health outcomes for older adults [62]. The limited past research on disordered eating correlates among older adult populations has focused on constructs relevant to eating disorders in younger populations (e.g., body appearance concerns, maladaptive perfectionism; [4]), with recent data indicating BE is correlated with BMI, negative mood, worry, and frequency of consuming nutritious foods [55]. This study is the first to examine health indices, particularly those important in geriatrics (e.g., physical functioning, social isolation, sleep, depression), in the context of BE among older adult women. Based on current findings using only online surveys, more research is needed to better understand the transactional relations between BE and healthspan indicators in older adults using a combination of interview and medical assessments within a longitudinal design. The simplest way to accomplish this is by adding BE assessments to existing longitudinal studies in older women.

Both limitations and strengths of the study should be considered when drawing conclusions. One limitation is the exclusive use of self-report measures, which may have resulted in memory/recall biases, or in the over- or under-reporting of symptoms/behaviors. In this vein, without clinical diagnostic interviews to evaluate objectively the amount of food consumed during self-reported binge episodes (e.g., the Eating Disorders Examination; [63]), participants could have been endorsing objective BE, subjective BE, or even general loss-of-control eating (e.g., grazing, feeling out of control of eating behaviors or choices). Thus, findings must be interpreted in light of this methodological limitation. Future research should consider more comprehensive evaluations of binge eating behaviors, including the full spectrum of binge eating and related conditions, in order to enhance validity of frequency cutoffs and categorizations. Yet, our data suggest that older women’s self-perception of experiencing BE—defined as eating “extremely large amounts of food at one time and [feeling] that [their] eating was out of control at that time”—was linked with poorer health indices in this sample. This finding is consistent with past research among younger adult populations suggesting that eating loss of control, regardless of food amount consumed, was associated with poorer psychological health (e.g., depression) [64, 65]. Future research to explicate health comorbidities or consequences of BE behaviors (subjective and/or objective) versus clinical binge eating disorder among older adult populations is warranted.

Additional limitations include non-random sampling and use of an online survey, resulting in a demographically homogenous sample and limiting generalizability. Specifically, older adult women living without internet access or limited technological proficiency may have been missed by this sampling procedure and survey methodology. Although we did use in-person recruitment as well (e.g., local senior centers, Community Advisory Board meetings), these avenues were limited to our local community in South Texas. The majority of our sample reported non-Hispanic White race/ethnicity, which limits generalizability of findings. Furthermore, our sampling procedure elicited a high rate of respondents with higher levels of education and income; thus, we are unable to generalize findings to older adults with more diverse backgrounds, education levels, and living circumstances. Additionally, the lack of longitudinal data limits the ability to examine temporal relations. It is possible that problems are a consequence, a correlate, or a cause of BE. Future research is needed to discern these patterns because each association has unique clinical implications regarding public health significance, treatment, and prevention efforts. Similarly, research is warranted to disentangle the trajectories of disordered eating patterns across the lifespan (i.e., chronic course, remission-relapse, or later-life onset). Finally, future work should include men and women, more demographically diverse samples, and expand examination to variables that may play a unique role in BE in older populations (e.g., menopause symptoms).

Strengths of the current study include the novel use of health outcomes that are of particular importance in the geriatrics field (e.g., functional status, quality of life, depression; [62]) in relation to BE symptomatology among an understudied population of older adult women. Indeed, findings indicate that older adult women with BE experience common comorbidities, QOL impairment, and wellness behaviors as younger individuals with BE. Additionally, we did not use targeted recruitment for women with eating concerns, which could have artificially inflated the rates of BE as well as the relations between BE and health indices. Indeed, the consistency in observed rate of weekly BE in our sample with past research [7, 55] is a strength of this study. Measures demonstrated strong psychometric properties in our sample, supporting participants attended to measures during online assessment, and this was verified through data quality checks. We employed two measures of BE that demonstrated consistent findings, lending confidence to our results.

In conclusion, our data suggest that BE occurring weekly or more represents a clinically meaningful threshold signifying increased problems with health behaviors, mental health, and health-related QOL among older adult women. Notably, BE was associated with poorer healthspan indices while controlling for BMI; thus, differential health outcomes between BE groups was not exclusively due to elevated BMI status in the BE group. Incorporation of BE screening into routine assessment procedures among geriatrics health practices would offer a brief avenue to potentially uncover an unmet need for clinical care among older adults. Improved identification of and treatment for BE could prevent further worsening of healthspan associated with this prevalent form of overnutrition among older adult women.

## Data Availability

The dataset generated and/or analyzed during the current study is not publicly available but is available from the corresponding author on reasonable request.

## Abbreviations

BE: Binge eating
BMI: Body mass index
DSM-5: Diagnostic and statistical manual for mental disorders, 5th edition
IBR: Institutional Review Board
VA-BES: Veterans affairs-binge eating screener
BES: Binge eating scale
EBQ: Eating behaviors questionnaire
PSQI: Pittsburgh sleep quality index
PASE: Physical activity scale for the elderly
PANAS-X: Positive and negative affect schedule
QOL: Quality of life
SF-36: RAND 36-item short form health survey
CES-D: Center for epidemiologic studies-depression scale
GAI: Geriatric anxiety inventory form
PANAS-X: Positive and negative affect schedule
BAS: Body appreciation scale
DM: Diabetes mellitus
