# Correction: the clinical significance of binge eating among older adult women: an investigation into health correlates, psychological wellbeing, and quality of life

**DOI:** 10.1186/s40337-023-00928-3

**Published:** 2023-11-15

**Authors:** Lisa Smith Kilpela, Victoria B. Marshall, Pamela K. Keel, Andrea Z. LaCroix, Sara E. Espinoza, Savannah C. Hooper, Nicolas Musi

**Affiliations:** 1 Barshop Institute, UT Health San Antonio, San Antonio, TX USA; 2 ReACHCenter, UT Health San Antonio, San Antonio, TX USA; 3 South Texas VA Health System, Audie Murphy Veterans Hospital, San Antonio, TX USA; 4 Department of Psychology, Florida State University, Tallahassee, FL USA; 5 Herbert Wertheim School of Public Health and Longevity Science, University of California San Diego, San Diego, CA USA

**Correction: Journal of Eating Disorders (2022) 10:97** 10.1186/s40337-022-00621-x

The original article [1] has incomplete funding information. The sponsorship from Takeda Pharmaceutical Australia to cover the Article Processing Charge was not mentioned in the Funding note. The correct Funding note is as follows.

**Funding**

This work was supported by the National Institute on Aging (NIA) [K76AG060003-A1]. The article has received sponsorship from Takeda Pharmaceutical Australia to cover the Article Processing Charge.

The original article [1] has been corrected.
